# Triple-Band Surface Plasmon Resonance Metamaterial Absorber Based on Open-Ended Prohibited Sign Type Monolayer Graphene

**DOI:** 10.3390/mi14050953

**Published:** 2023-04-27

**Authors:** Runing Lai, Pengcheng Shi, Zao Yi, Hailiang Li, Yougen Yi

**Affiliations:** 1 Joint Laboratory for Extreme Conditions Matter Properties, Southwest University of Science and Technology, Mianyang 621010, China; lrn3167160774@163.com (R.L.);; 2 School of Chemistry and Chemical Engineering, Jishou University, Jishou 416000, China; 3 Key Laboratory of Microelectronic Devices & Integrated Technology, Institute of Microelectronics, Chinese Academy of Sciences, Beijing 100029, China; 4 College of Physics and Electronics, Central South University, Changsha 410083, China

**Keywords:** graphene, tripe-band perfect absorption, polarization independence, incident angle insensitivity, tunable

## Abstract

This paper introduces a novel metamaterial absorber based on surface plasmon resonance (SPR). The absorber is capable of triple-mode perfect absorption, polarization independence, incident angle insensitivity, tunability, high sensitivity, and a high figure of merit (FOM). The structure of the absorber consists of a sandwiched stack: a top layer of single-layer graphene array with an open-ended prohibited sign type (OPST) pattern, a middle layer of thicker SiO_2_, and a bottom layer of the gold metal mirror (Au). The simulation of COMSOL software suggests it achieves perfect absorption at frequencies of *f*_I_ = 4.04 THz, *f*_II_ = 6.76 THz, and *f*_III_ = 9.40 THz, with absorption peaks of 99.404%, 99.353%, and 99.146%, respectively. These three resonant frequencies and corresponding absorption rates can be regulated by controlling the patterned graphene’s geometric parameters or just adjusting the Fermi level (*E_F_*). Additionally, when the incident angle changes between 0~50°, the absorption peaks still reach 99% regardless of the kind of polarization. Finally, to test its refractive index sensing performance, this paper calculates the results of the structure under different environments which demonstrate maximum sensitivities in three modes: *S*_I_ = 0.875 THz/RIU, *S*_II_ = 1.250 THz/RIU, and *S*_III_ = 2.000 THz/RIU. The FOM can reach FOM_I_ = 3.74 RIU^−1^, FOM_II_ = 6.08 RIU^−1^, and FOM_III_ = 9.58 RIU^−1^. In conclusion, we provide a new approach for designing a tunable multi-band SPR metamaterial absorber with potential applications in photodetectors, active optoelectronic devices, and chemical sensors.

## 1. Introduction

In recent years, a new development has emerged in the field of artificial absorbers—the preparation of perfect metamaterial absorbers (PMAs). This process utilizes surface plasmon resonance (SPR), which involves the absorption of incident electromagnetic waves by electrons on the surface and interface of the metamaterial, thereby reducing the reflection [1,2]. The incident wave couples with the electromagnetic component, surpassing the conventional optical diffraction limit and enhancing the local electromagnetic field, ultimately achieving perfect absorption. PMAs have a wide range of applications in optical sensing, optical stealth, light detection, photothermal conversion, photocatalysis, and many other fields [3,4,5,6,7,8,9,10,11,12]. However, the traditional SPR structure is usually fixed and lacks the ability to dynamically adjust the resonance frequency and absorption rate. In addition, the sensitivity also has a connection with the polarization and incident angle. These factors limit the flexibility of a PMA in practical applications [12,13,14,15,16,17]. Therefore, an appropriate metamaterial must be employed to realize a perfect SPR metamaterial absorber that is tunable and incident angle insensitive.

In order to achieve tunability in devices, many researchers have employed graphene [18], vanadium dioxide (VO_2_) [19], and Dirac semimetals [20]. Among these materials, graphene stands out due to its extremely high conductivity and carrier mobility, which provides a full-spectrum response to terahertz waves and strong surface plasmon resonance [21,22,23]. Moreover, the Fermi level of graphene can be precisely controlled through external voltage [24], while materials such as VO_2_ are difficult and expensive to control due to their abrupt phase transitions [25]. Furthermore, graphene exhibits a fast response throughout the entire terahertz range [26], enabling graphene materials to achieve multiple perfect absorption peaks in the terahertz range with improved spacing between resonant frequencies and modulation bandwidth. On the other hand, Dirac semimetal materials are a new type of 3D material [27] that has been studied less compared to graphene. The experimental equipment required for their preparation is limited and demanding, making actual production difficult, and their response to terahertz frequencies is low, making it challenging to achieve absorption rates above 99% and narrow spectral bandwidths between multiple peaks. Based on these characteristics, graphene-based absorbers can achieve perfect absorption at specific resonant frequencies. Therefore, the design of graphene-based absorbers is a popular topic.

Among many surface plasmon metamaterial absorbers, narrowband perfect absorbers and their refractive index sensing properties have been a popular topic of research. For instance, in 2017, Chen et al. proposed a single peak absorber with an absorption of 99.51% at 2.71 THz. Later, dual-frequency and wide-band absorption were achieved by simply stacking two layers of different geometric sizes of graphene metasurfaces, resulting in absorption rates of 98.94% at 1.99 THz and 99.1% at THz [28]. Two years later, Yan et al. had a discussion on a tunable single-mode absorber about the influence of changes in the Fermi level concerning resonant frequencies. The maximum absorption rate can reach 99.99% at *E_F_* = 0 eV [29]. More recently, in 2022, Zhu et al. designed an absorber that had a single-band and dual-band. The single-band absorption is 99.30% at 16.0 THz, while the dual-band absorption could reach 94.56% at 11.4 THz and 99.11% at 26.2 THz, respectively [30]. However, most studies have focused on only one or two narrowband metamaterial absorbers, and there is little discussion about the ideal narrowband perfect absorbers for multi-band applications. This is due to strict limitations on the design and the difficulty of implementation with simple structures.

This paper proposes a novel three-mode metamaterial SPR absorber based on an open-ended prohibited sign type (OPST) patterned graphene. Compared with other structures, the proposed structures possess the novelty of simple graphene patterns, multi-band absorption, stable dielectric material properties, and flexible geometric parameters. The structure comprises a periodic graphene array with an OPST pattern on the top, followed by a thick silica (SiO_2_) spacer layer and a metallic gold (Au) mirror at the bottom. Using COMSOL Multiphysics simulation software, we were able to calculate and observe perfect absorption rates of 99.404%, 99.353%, and 99.146% at 4.04 THz, 6.76 THz, and 9.40 THz, respectively, independent of polarization. Then, the principle of perfect absorption of the absorber was carefully studied by analyzing the local electric field intensity distribution and mapping the equivalent impedance matching diagrams. Additionally, we demonstrated tunability by changing *E_F_* while keeping the structural parameters fixed. To test the sensor performance of the device, we changed the surrounding refractive index to achieve a maximum FOM and sensitivity of 9.58 RIU^−1^ and 2.000 THz/RIU, respectively. Compared to other graphene SPR metamaterial absorbers, this offers more flexible geometric parameters and sensitive sensing properties; thus, promoting greater diversity in the design of graphene-based metamaterial absorbers and providing new design inspiration. Therefore, we believe that this new type of SPR metamaterial absorber has potential applications in the field of active optoelectronic devices, modulators, refractive index sensors, and detectors.

## 2. Structure and Design

**Figure 1:**
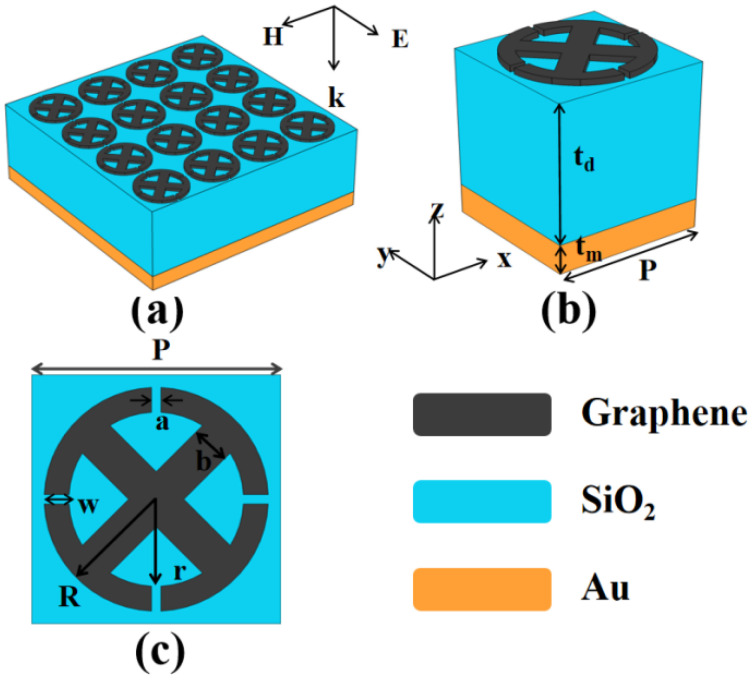
(**a**) Overall periodic structure of the OPST graphene metamaterial absorber, (**b**) the single unit structure, and (**c**) a view of the OPST patterned graphene, where *P* = 4 μm, *a* = 0.15 μm, *b* = 0.63 μm, *r* = 1.4 μm, *R* = 1.8 μm, *w* = *R* − *r* = 0.4 μm, *t_d_* = 4.6 μm, and *t_m_* = 1 μm.

As shown in Figure 1a, the whole periodic structure of the metamaterial absorber consists of a high sensitivity and multi-band open-ended prohibited sign type (OPST) single-layer patterned graphene array with certain geometric parameters. It comprises a sandwich-stacked structure of graphene layer deposited on SiO_2_/Au substrates. We utilized COMSOL Multiphysics software to simulate the physical process of absorption using the finite element method (FEM) [31,32]. The simulation involved a SiO_2_ dielectric layer with a refractive index of 1.97, an Au layer with a dielectric constant of 1.00, and a graphene layer considered a homogeneous medium with a thickness of *t_g_* = 1 nm [33].

A 3D diagram of the absorber unit with a structure is shown in Figure 1b with a structure period of *P* = *P_x_* = *P_y_* = 4 μm. The thickness of the SiO_2_ and Au layer is *t_d_* = 4.6 μm and *t_m_* = 1 μm, respectively. In addition, Figure 1c portrays a vertical view of the OPST graphene, which illustrates more details on geometric parameters. The array comprises two parts: a ring with four openings and a cross-like structure. As for the open-ended ring structure, the inner radius is *r* = 1.4 μm, the outer radius is *R =* 1.8 μm, *w* = *R − r* = 0.4 μm, and the opening width is *a* = 0.15 μm. The width of the cross-like structure is *b* = 0.63 μm, and its end curvature is equal to 1/*r*. In the X and Y directions in COMSOL software, periodic boundary conditions are used. PML (perfect matching layers) are set in the Z direction. The incident frequency is set at 1~10 THz. The total conductivity of graphene is defined as *σ_g_* = *σ*_intra_ + *σ*_inter_ [34], where *σ*_intra_ represents intra-band conductivity, while *σ*_inter_ is inter-band conductivity.

They can be described (simplified) by the Kubo formula [35]:

(1) $$\sigma_{\mathrm{intra}}=\frac{ie^{2}k_{B}T}{\pi \hbar^{2}(\omega +i\tau^{-1})}\left\{\frac{E_{F}}{k_{B}T}+2\ln\left[\exp(-\frac{E_{F}}{k_{B}T})+1\right]\right\}$$

(2) $$\sigma_{\mathrm{inter}}=\frac{ie^{2}}{4\pi \hbar^{2}}\ln\left[\frac{2\left|E_{F}\right|-\hbar (\omega +i\tau^{-1})}{2\left|E_{F}\right|+\hbar (\omega +i\tau^{-1})}\right]$$

Here, *ω* is the incident angular frequency, *e* is the charge of a single electron, *ħ* is the reduced Planck constant, *k_B_* is the Boltzmann constant, *T* is the surrounding temperature, *E_F_* is the Fermi level of graphene, and *τ* is the relaxation time of graphene. Due to the Pauli exclusion principle, *E_F_* ≫ *ħω* in the terahertz band, causing the surface conductivity of graphene to be mainly determined by the intra-band contribution. As a result, the *σ*_inter_ term can be ignored, simplifying the *σ*_intra_ to Drude conductivity, and the *σ_g_* can be expressed as [36,37]:

(3) $$\sigma (\omega )=\frac{ie^{2}\left|E_{F}\right|}{\pi \hbar^{2}(\omega +i\tau^{-1})}$$

From Equation (3), we can infer that *σ*(*ω*) can be dynamically adjusted by changing *E_F_* and *τ* to get certain resonant frequencies. In terms of *A* = 1 − *R* − *T*, absorption *A* equals 1 minus reflectance *R* and transmittance *T*. Furthermore, it approaches 1 when *R* and *T* are small enough [38,39,40]. Au is an excellent conductor, and when electromagnetic waves hit its surface, they rapidly diminish in strength. The skin depth of Au is defined as the distance at which the wave’s amplitude has decayed to 1/*e* of the value at the surface:

(4) $$e^{-\alpha \delta }=1/e\Rightarrow \delta =\frac{1}{\alpha }=\sqrt{\frac{2}{\omega \mu \sigma }}=\frac{1}{\sqrt{\pi f\mu \sigma }}$$

In this study, the range of *f* is 10^12^~10^13^ Hz (1~10 THz), with *μ* = *μ*_0_ = 4*π* × 10^−7^ N/A^2^ and *σ* = 4.56 × 10^7^ S/m of Au. Therefore, the skin depth can be calculated as 7.4531 × 10^−2^~2.3569 × 10^−2^ μm, which is significantly smaller than the thickness set in the simulation of *t_m_* = 1 μm, indicating that the thickness of the Au layer is sufficient to block the propagation of electromagnetic waves and ensure *T* is negligible (*T* = 0). When SPR occurs, *R* = 0, indicating that perfect absorption is theoretically achieved.

**Figure 2:**
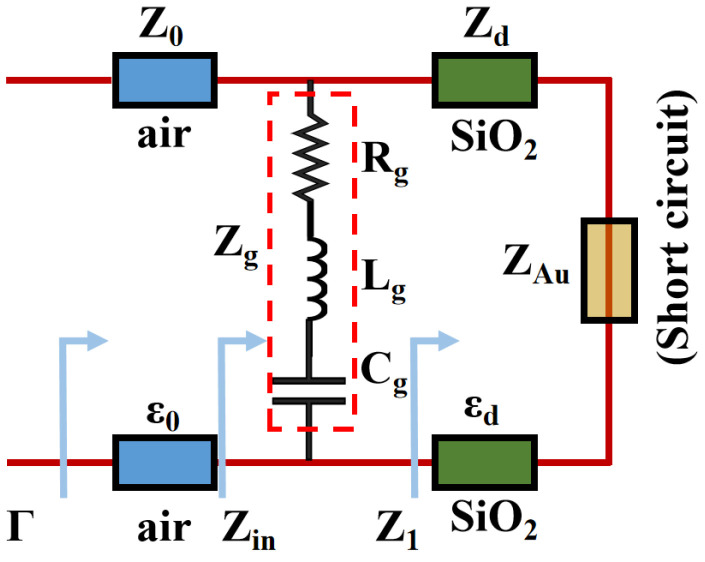
The equivalent circuit of the proposed SPR metamaterial absorber.

In order to demonstrate the proposed structure an equivalent circuit is utilized, as shown in Figure 2. It provides a clear visualization of how the components of the structure interact and contribute to the overall performance. In this model, the Au layer is considered a short circuit so Z_Au_ is negligible [41].

In this model, graphene is equivalent to a resistor, an inductor, and a capacitor. The parameters in the figure are expressed as follows:

(5) $$\left\{\begin{array}{l} Z_{1}=jZ_{d}\tan(k_{d}t_{d})\\ Z_{d}=\sqrt{\mu_{0}/\varepsilon_{0}\varepsilon_{d}}\\ k_{d}=2\pi f\sqrt{\varepsilon_{0}\varepsilon_{d}\mu_{0}} \end{array}\right.$$

(6) $$Z_{g}=R_{g}+jX_{g}=R_{g}+j(2\pi fL_{g}-\frac{1}{2\pi fC_{g}})$$

**Figure 3:**
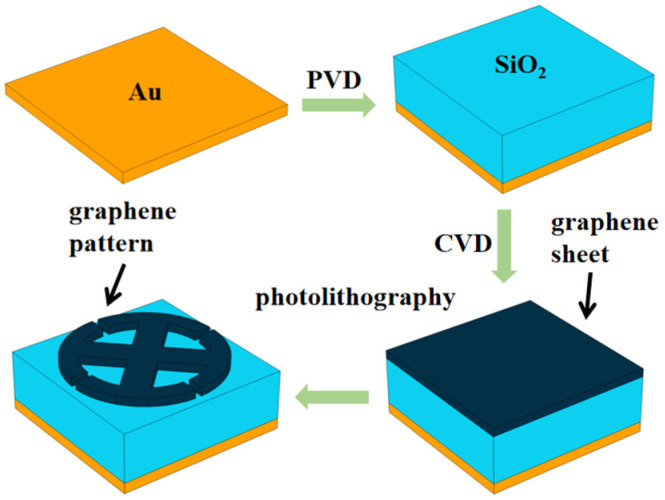
The fabrication program of the proposed metamaterial absorber.

(7) Zin=Z1·ZgZ1+ZgΓ=Re(Zin)−Z0Re(Zin)+Z0Z0=μ0ε0=120π

where the calculation of *R_g_*, *L_g_*, and *C_g_* of graphene can be referenced in [42]. The absorption coefficient of the absorber can be described using reflection efficiency Γ: *A* = 1 − Γ^2^. Thus, perfect absorption can be achieved when Re(*Z_in_*) = *Z*_0_ (intrinsic impedance in free space), which is also discussed in the next section with effective impedance matching theory. The proposed metamaterial absorber is shown in Figure 3.

In actual fabrication, the first step is to clean a silicon substrate with acetone and isopropanol alcohol. Then, it is dried with high-purity compressed nitrogen. Next, a gold ground plane is coated onto the substrate using electron beam evaporation (a type of physical vapor deposition technique, PVD) [43] at room temperature. A SiO_2_ layer is then spin-coated onto the gold plane, and its thickness is calibrated with a stylus profilometer (Dektak XT—Bruker, Bilrika, America) to ensure it reaches the desired thickness. The final step is to grow a graphene layer on a copper catalyst using chemical vapor deposition (CVD). Once the graphene layer is grown, photolithography is used to create the OPST pattern.

## 3. Results and Discussion

**Figure 4:**
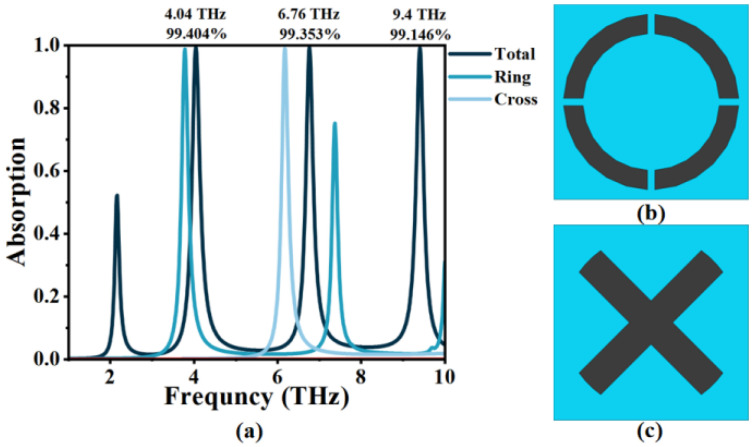
(**a**) The absorption of the open-ended prohibited sign type graphene absorber and its two sub-structures; (**b**) a view of the open-ended ring-type graphene absorber; and (**c**) a view of the cross-like type graphene absorber.

Figure 4a suggests the total absorption (the black line) of the OPST graphene absorber, which is formed by combining an open-ended ring and a cross-like shape. It can be seen that the OPST graphene absorber exhibits three narrow absorption peaks. Figure 4b,c shows its two sub-structures. Evidently, the total absorption is significantly improved by combining these two types, especially at 6.76 THz. The absorber achieves perfect absorption at frequencies *f*_I_ = 4.04 THz, *f*_II_ = 6.76 THz, and *f*_III_ = 9.40 THz, with efficiencies of 99.404%, 99.353%, and 99.146%, which is named Mode I–III. The OPST graphene will generate surface plasmon in contact with the incident wave, and wavelengths of the three modes are strongly confined by the graphene. The incident wave coincides with the frequency of the surface free electrons, resulting in SPR [44,45]. This then excites plasma oscillations, resulting in strong absorption of energy and the achievement of perfect absorption.

**Figure 5:**
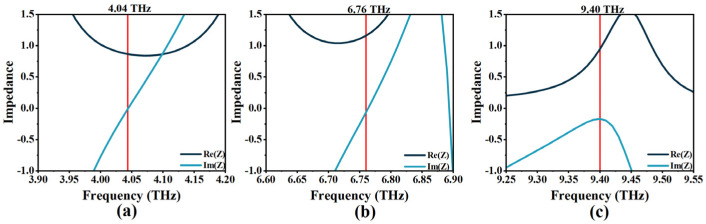
(**a**–**c**) The effective impedance matching diagrams of Mode I–III of the OPST graphene absorber.

To obtain clear proof of perfect absorption, we used the effective impedance matching principle of an ideal electromagnetic metamaterial absorber. The corresponding results are shown in Figure 5. It displays the changes between the impedance and the incident frequencies of Mode I–III. This relationship is based on the equivalent impedance theory formula [46]:

(8) $$Z=\sqrt{\frac{{\left(1+S_{11}\right)}^{2}-S_{21}{}^{2}}{{\left(1-S_{11}\right)}^{2}-S_{21}{}^{2}}}$$

The equivalent impedance, denoted by *Z*, is related to the scattering parameters *S*_11_ and *S*_21_, which correspond to reflectance and transmittance, respectively. According to the equivalent impedance theory formula, perfect absorption occurs when the equivalent impedance is matched with free space impedance *Z*, leading to a significant decrease in reflection (*S*_11_ = 0). This is achieved when the real part (Re(Z)) is close to One while the imaginary part (Im(Z)) is close to Zero. Through a comprehensive analysis of Figure 4 and Figure 5, we can find that these three resonant frequencies of the OPST graphene absorber are indeed perfectly matched, as described by the theory.

**Figure 6:**
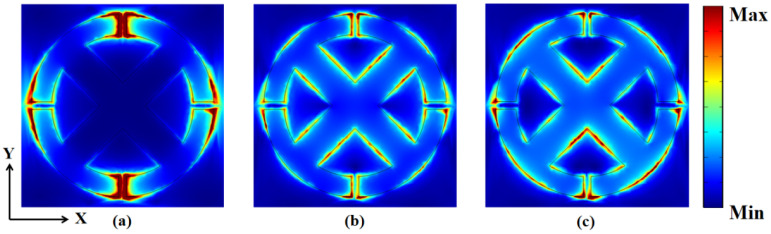
The electric field distribution on surface in the X-Y plane at three different incident frequencies: (**a**) 4.04 THz, (**b**) 6.76 THz, and (**c**) 9.40 THz.

In order to further study the three modes of the principle of perfect absorption [36], the electric field monitors are set at *f*_I_ = 4.04 THz, *f*_II_ = 6.76 THz, and *f*_III_ = 9.40 THz, respectively, and we obtained the cross-sectional electric field distribution in the X-Y direction, as shown in Figure 6. The intensity values of the electric field are represented by the color bar presented on the right side of the figure, with stronger values indicated by warmer colors (e.g., red). In Figure 6a, the electric field is mainly concentrated at the four openings of the ring. At 6.76 THz in Figure 6b, an electric field is also excited at the straight edge of the cross and the edge on the outermost circle, but weakened at the four openings. In Figure 6c, the electric field is mainly excited at the edge of the four openings, the outermost circle, and near the intersection of the cross. These three cases can be attributed to the coupling of the vibration frequency of the patterned OPST graphene layer with waves at these three frequencies, providing electric dipole resonance and greatly consuming incident energy [47]. Thus, the absorber achieves a perfect match with the free-space impedance in the three resonance frequency bands. Moreover, the incident waves are perfectly absorbed eventually.

**Figure 7:**
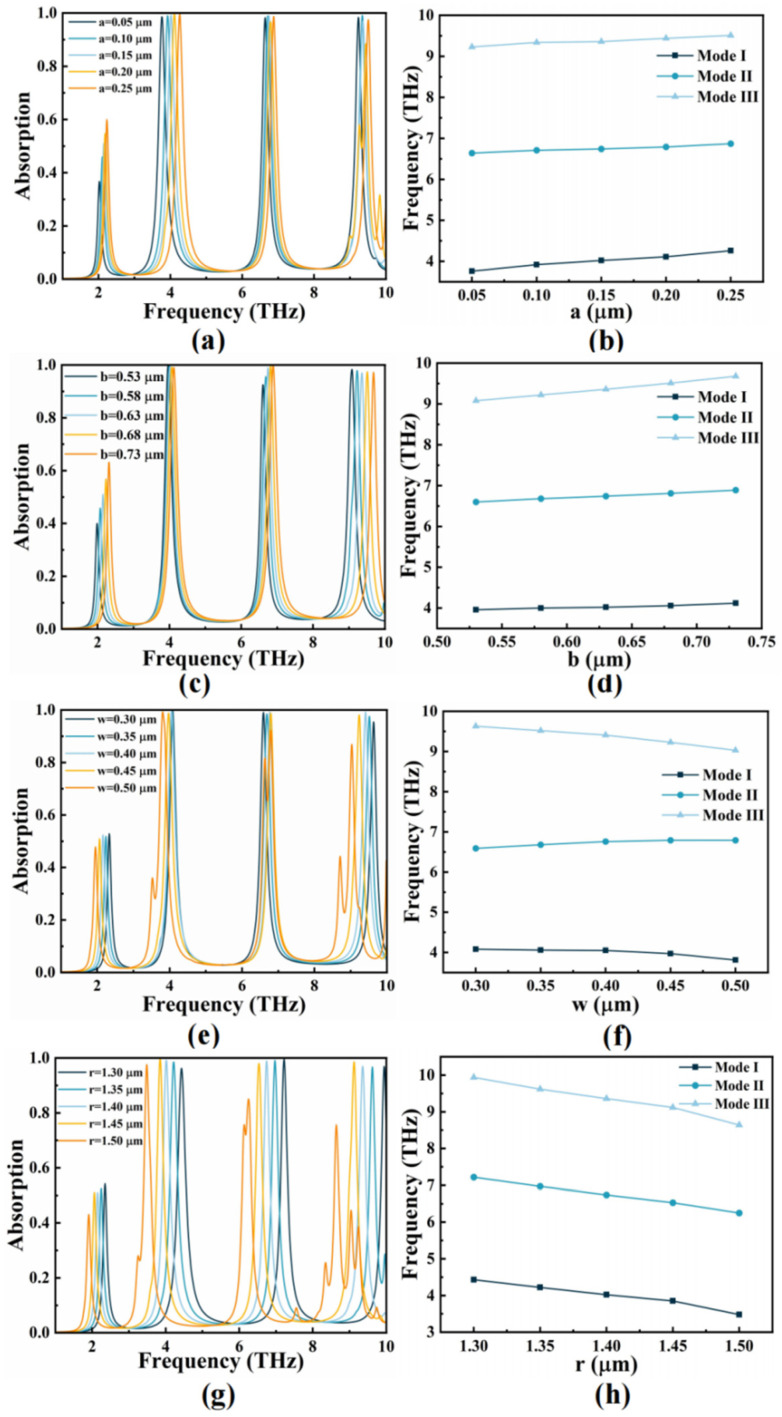
The changes in absorption and resonant frequency for each resonance mode as different geometric parameters are altered, while keeping the remaining parameters constant; (**a**,**c**,**e**,**g**) demonstrate the variations in absorption for each mode, while (**b**,**d**,**f**,**h**) exhibit the frequency shift in the resonant peaks.

Furthermore, by using the control variable method, we studied the effect on the absorption by adjusting the geometric parameters [48], the opening width *a*, the cross-width *b*, the ring width *w*, and the inner radius *r*. The corresponding results are suggested in Figure 7. Figure 7a,c,e,g displays the changes in absorption efficiencies of each resonance mode, and Figure 7b,d,f,h shows the corresponding shift of the resonant frequency. Figure 7a demonstrates that increasing parameter *a* from 0.05 μm to 0.25 μm enhances the absorption efficiency of Mode I but causes the absorption efficiency of other modes to initially increase and then decrease. This trend is attributed to the fact that parameter *a*, which refers to the opening width of the ring structure, affects the local electromagnetic fields of the three modes, as shown in Figure 6. Additionally, the resonance frequencies depicted in Figure 7b are blue-shifted, particularly in Mode I, indicating that parameter *a* mainly influences its resonance frequency. When *b* varies from 0.53 μm to 0.73 μm, the absorption efficiency of Mode I and Mode III barely changes (relative to Mode II), as demonstrated in Figure 7c. This is because the local electromagnetic field distribution in Mode I and III is almost unaffected by *b*, as it is not on the straight edge of the cross. The shift in the resonant frequencies of the three modes due to the change in parameter *b* shown in Figure 7d causes a blue shift, with the most significant change occurring in Mode III. In Figure 7e, changing parameter *w* does not significantly affect the absorption efficiencies of Mode I and II, but it noticeably impacts the absorption rate and resonance frequency of Mode III, resulting in a significant red shift. This effect can be attributed to the increase in the edge length of the outermost circle due to the increase in *w*, as *R* = *r* + *w* and *C* = 2*πR*. Consequently, the distribution region of the plasma broadens, as evident in Figure 6, and the resonance frequency of Mode III gradually decreases. The same phenomenon can be observed in Figure 7g,h when parameter *r* (or *R*) is changed, with Mode I and II also exhibiting a red shift. However, the pattern size of OPST graphene should not be too large, as this can cause it to couple to adjacent graphene arrays and affect the resonance frequency.

**Figure 8:**
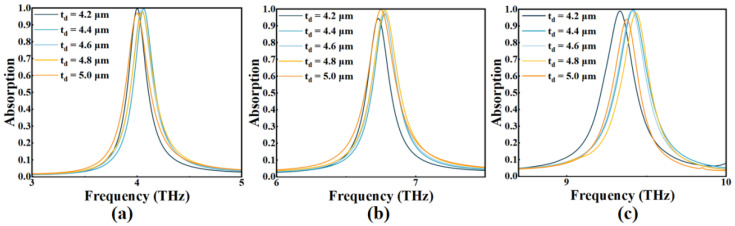
The absorption of three modes (**a**–**c**) when *t_d_* varies 4.2~5.0 μm.

In addition, we also discussed the determination of the thickness of the dielectric layer as shown in Figure 8. We can find that the thickness of SiO_2_ (*t_d_*) has an effect on the absorption of three mods, which is consistent with the expectation of theoretical derivation (Equations (5) and (7)). When *t_d_* = 4.6 μm, the absorption of Mode I–III could remain above 98.56% with the highest average value.

**Figure 9:**
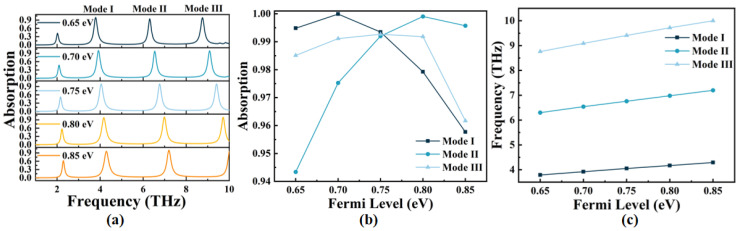
(**a**) The absorption spectrum when the geometric parameters are fixed with *E_F_* in range of 0.65~0.85 eV; (**b**,**c**) the absorption of the absorber and frequency shifts corresponding to Mode I–III when *E_F_* changes.

In practical applications, the dynamic tuning ability of absorbers is crucial, especially when the structural parameters are fixed. Figure 9 illustrates the change in the absorber’s absorption spectrum when the Fermi level (*E_F_*) of graphene is altered while keeping the structural parameters constant. *E_F_* is given by [49]:

(9) $$E_{F}=V_{f}\sqrt{\frac{\pi \varepsilon_{0}\varepsilon_{r}V_{g}}{e_{0}t_{d}}}$$

where *V_f_* is Fermi velocity, *ε*_0_ and *ε_r_* are the dielectric constant in a vacuum and relative dielectric constant, *V_g_* is the applied voltage, which can be controlled by altering the grid voltage or chemical doping, *e*_0_ is the amount of electron charge, and *t_d_* is the thickness of the dielectric layer.

As shown in Figure 9a,c, when *E_F_* increases from 0.65 eV to 0.85 eV, the resonance frequencies of Mode I–III suggest a blue shift. Due to how the resonance frequency *ω* of the metamaterial absorber is related to its capacitance *C*, when *E_F_* increases, *C* increases at the same time, leading to an increase in the resonance frequency. This phenomenon can also be explained in terms of the formula for resonant wavelengths: *λ*_res_ = *α* + *β* × *n*_sp_, where *n*_sp_ represents the effective refractive index of graphene, while *α* and *β* are coefficients that have close connections with the patterned graphene’s geometry and the surrounding dielectric properties [50]. Thus, as *E_F_* increases, *n*_sp_ decreases, causing *λ*_res_ to decrease eventually. Consequently, the resonance frequency increases, resulting in a blue shift. For Mode I, the resonance frequency blue shift is in the range of 3.79~4.29 THz, with the highest absorption of 99.99% at *E_F_* = 0.70 eV. For Mode II, the resonance frequency blue shift ranges from 6.30 THz to 7.20 THz, with the highest absorption rate of 99.91% at *E_F_* = 0.80 eV. Finally, for Mode III, the resonance frequency blue shift ranges from 8.76 THz to 10 THz (the maximum range is limited to 10 THz in this simulation when *E_F_* = 0.85 eV), and the maximum absorption rate is 99.27% at *E_F_* = 0.75 eV. The tuning sensitivity of the three resonant modes is 2.5 THz/eV, 4.5 THz/eV, and 6.2 THz/eV (up to 10 THz), respectively, indicating that the OPST graphene absorber exhibits excellent tunability. In other words, a slight change in *E_F_* can cause a significant shift in resonant frequency. Figure 9b demonstrates that the absorption of Mode I–III changes as *E_F_* varies. At *E_F_* = 0.75 eV, the average absorption of the three peaks reaches a maximum of 99.27%, suggesting that setting *E_F_* to 0.75 eV is a more balanced choice. In conclusion, the absorption and resonant frequencies of the OPST graphene absorber can be dynamically adjusted solely by altering *E_F_* without modifying its structural parameters, which is highly advantageous in practical applications.

**Figure 10:**
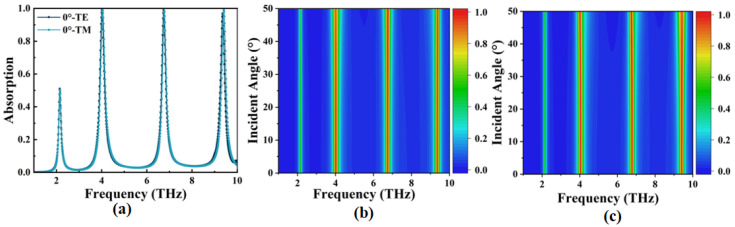
(**a**) The absorption when the incident angle is 0°; (**b**,**c**) the absorption when the incident angle is in the range of 0~50° under TE and TM polarizations, respectively.

We investigated the response characteristics of the OPST graphene absorber to changes in the polarization mode and incident angle of electromagnetic waves. The results are shown in Figure 10. In Figure 10a, we initially assumed that electromagnetic waves were incident vertically on the absorber’s surface under two polarizations (TE and TM), that is, when the incident angle is 0°. The two absorption diagrams are highly consistent, suggesting that the OPST graphene absorber is insensitive to the two polarizations due to the center symmetry of its surface geometry. Next, we gradually increased the incident angle from 0° to 50° under TE and TM polarizations, respectively, and we obtained the results shown in Figure 10b,c. It can be seen that the absorption spectra do not change significantly with the increasing incident angle regardless of the polarization mode, demonstrating the incident angle insensitivity of the OPST graphene absorber. Due to the highly symmetric design of the graphene pattern and the fact that the localized surface plasmon resonance (LSPR) wavelength of the graphene nanostructure is smaller than the vacuum wavelength of the incident light [51,52,53], it exhibits strong local surface plasmon resonance when the incident light angle is between 0~50° while maintaining the symmetry of the structure. However, under TE polarization, the influence of the incident angle on Mode I absorptivity was greater than that of the other two modes, decreasing from 99.18% at 0° to 91.78% at 50°. Similarly, Mode III absorption decreased from 99.25% at 0° to 90.80% at 50° under TM polarization. This is because, at these two resonant frequencies, the incident area decreases with the increasing incident angle, which easily leads to weakened plasmon resonance intensity and decreased absorption [54,55]. All discussions in this paper are based on TE polarization patterns unless otherwise noted. In summary, the OPST graphene absorber is polarization and incident angle insensitive under 0~50°. In practical applications, a concave structure can be designed above the absorber’s surface to couple with the incident wave at a specific incident angle, achieving perfect absorption.

**Figure 11:**
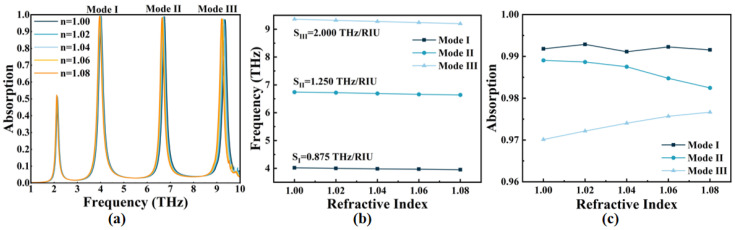
(**a**) Absorption of Mode I–III with different *n*; (**b**) the relation between resonant frequency and *n* of Mode I–III with corresponding sensitivities; (**c**) the absorption of each resonance mode varies with *n*.

Finally, we examined the ambient refractive index *n* sensing ability of the OPST graphene absorber, as presented in Figure 11. As *n* ranges from 1.00~1.08, the three resonant frequencies corresponding Mode I–III exhibit a red shift, with a range of Δ*f* being 3.95~4.02 THz, 6.64~6.74 THz, and 9.20~9.36 THz, respectively. The corresponding sensitivity *S* is calculated as [56,57]:

(10) $$S=\frac{\Delta f}{\Delta n}$$

Here, Δ*n* is 1.08 − 1.00 = 0.08 in this study, and the sensitivity of the three modes is represented as *S*_I_ = 0.875 THz/RIU, *S*_II_ = 1.250 THz/RIU, and *S*_III_ = 2.000 THz/RIU, where RIU refers to refractive index unit. The high-frequency resonance frequency exhibits a stronger electric field density, making it more responsive to environmental changes. Therefore, the sensitivity at high resonant frequencies (e.g., Mode III) is higher, resulting in a larger red shift range. Moreover, the absorption of the three modes remains above 97%, even when the ambient refractive index changes.

**Figure 12:**
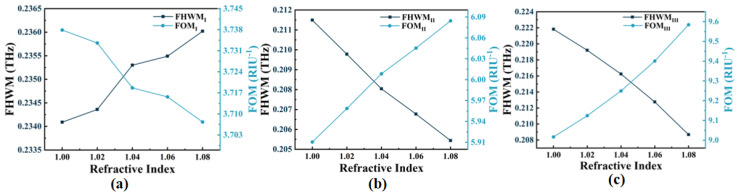
(**a**–**c**) The changes of FWHM and FOM of Mode I–III with *n* in the range of 1.00~1.08.

Figure 12 illustrates the variations in FWHM of the absorption in three modes and the figure of merit (FOM) in three modes. FOM represents the ratio of sensitivity to FWHM [58,59]:

(11) $$\mathrm{FOM}=\frac{S}{\mathrm{FWHM}}$$

Equation (11) states that the maximum FOM of the three modes is FOM_I_ = 3.74 RIU^−1^, FOM_II_ = 6.08 RIU^−1^, and FOM_III_ = 9.58 RIU^−1^, respectively. The findings indicate that the absorber possesses higher *S* and FOM and exhibits dynamic adjustment polarization independence and incident angle insensitivity characteristics, which have superior sensing performance and a broad range of potential applications. As listed in Table 1, the proposed absorber is compared with previous works. Evidently, the sensitivity in the refractive index sensing of our design has been significantly improved.

## 4. Conclusions

In summary, we designed a novel SPR metamaterial absorber that offers triple-mode perfect absorption, tunability, polarization independence, incident angle insensitivity, high sensitivity, and high FOM. It is a sandwich-stacked structure with a monolayer graphene periodic array featuring an open-ended prohibited sign type (OPST) pattern on the top, a thicker layer of SiO_2_ in the middle, and a Au mirror at the bottom. Compared with other structures, the proposed structures possess the novelty of simple graphene patterns, stable dielectric material properties, flexible geometric parameters, multi-band absorption, and high sensitivity. Based on the study in the COMSOL Multiphysics software, we conclude that the absorber achieves perfect absorption at *f*_I_ = 4.04 THz, *f*_II_ = 6.76 THz, and *f*_III_ = 9.40 THz, respectively, and exhibits angle insensitivity when the incident angle is less than 50°. Therefore, the absorber has potential application value in active optoelectronic devices. In this study, we explore the mechanism behind achieving perfect absorption in 1~10 THz using the effective impedance matching principle. By mapping the local electric field distribution in the X-Y plane, we gain a deeper understanding of the absorber’s operation. Furthermore, the effect of varying the geometrical parameters of the absorber and the Fermi level of graphene on the resonant frequency and absorption peak of the three resonant modes of the absorber is investigated. In addition, we examined the sensor performance by adjusting the ambient refractive index. The results suggest the maximum *S* and FOM can reach 2.00 THz/RIU and 9.58 RIU^−1^, respectively. These findings suggest that the proposed device has significant potential for use in photoelectric detection, chemical sensing, and related fields.

## Figures and Tables

**Table 1:** Comparison with the previous literature reports.

Reference	[60]	[61]	[62]	[63]	[64]	This Work
S (THz/RIU)(max)	1.57	1.12	1.84	0.875	2.1	2.00
FOM(RIU^−1^)(max)	24.5	50.59	N/A	26.51	7.03	9.58
Tunability	Yes	Yes	Yes	Yes	Yes	Yes

## Data Availability

Publicly available datasets were analyzed in this study. This data can be found here: [https://www.lumerical.com/ (accessed on 1 January 2020)].
